# Adherence to the 2018 World Cancer Research Fund/American Institute for Cancer Research Recommendations and Breast Cancer in the SUN Project

**DOI:** 10.3390/nu12072076

**Published:** 2020-07-13

**Authors:** Rocio Barrios-Rodríguez, Estefanía Toledo, Miguel Angel Martinez-Gonzalez, Inmaculada Aguilera-Buenosvinos, Andrea Romanos-Nanclares, José Juan Jiménez-Moleón

**Affiliations:** 1 Departamento de Medicina Preventiva y Salud Pública, Universidad de Granada, 18016 Granada, Spain; rbarrios@ugr.es (R.B.-R.); jjmoleon@ugr.es (J.J.J.-M.); 2 Consortium for Biomedical Research in Epidemiology and Public Health (CIBERESP), 28029 Madrid, Spain; 3 Instituto de Investigación Biosanitaria ibs.GRANADA, 18014 Granada, Spain; 4 Department of Preventive Medicine and Public Health, School of Medicine, University of Navarra, 31008 Pamplona, Spain; mamartinez@unav.es (M.A.M.-G.); iaguilera@alumni.unav.es (I.A.-B.); aromanos@alumni.unav.es (A.R.-N.); 5 Centro de Investigación Biomédica en Red Área de Fisiología de la Obesidad y la Nutrición (CIBEROBN), 28029 Madrid, Spain; 6 IdiSNA, Navarra Institute for Health Research, 31008 Pamplona, Spain; 7 Department of Nutrition, Harvard T.H. Chan School of Public Health, Boston, MA 02115, USA

**Keywords:** breast cancer, 2018 WRCF/AICR recommendations, SUN Project, cohort

## Abstract

A proportion of breast cancer cases are attributable to combined modifiable risk factors. The World Cancer Research Fund/American Institute for Cancer Research (WCRF/AICR) has recently updated the recommendations for cancer prevention and a standard scoring system has been published. The aim of this study was to evaluate the association between compliance with the 2018 WCRF/AICR cancer prevention recommendations (Third Expert Report) and the risk of breast cancer in the SUN (“Seguimiento Universidad de Navarra”) prospective cohort. Spanish female university graduates, initially free of breast cancer, were included (n = 10,930). An 8-item score to measure compliance to the recommendations was built: body fat, physical activity, consumption of wholegrains/vegetables/fruit/beans, “fast foods”, red/processed meat consumption, sugar-sweetened drinks consumption, alcohol intake, and breastfeeding. A stratified analysis was conducted according to menopausal status. A non-significant inverse association was observed for overall breast cancer. The inverse association became statistically significant for post-menopausal breast cancer after multivariable adjustment (hazard ratio for > 5 vs. ≤ 3 points = 0.27; 95% CI: 0.08-0.93). The results suggested that the possible inverse association with breast cancer was attributable to the combined effects of the different nutritional and lifestyle components.

## 1. Introduction

Breast cancer is the second most commonly diagnosed tumor around the world, representing 11.6% of the total cancer cases [1]. In women, it was the most frequently diagnosed cancer in both developed and developing countries in 2018 [2]. There were 2.1 million newly diagnosed female breast cancer cases, which accounted for almost 1 in 4 cancer cases among women [1]. In spite of the variability in breast cancer incidence across Europe, breast cancer is the leading cancer among women in every European country and it is the first overall cause of death from cancer among women [3].

Both modifiable and nonmodifiable risk factors may increase the risk of breast cancer [4]. Up to 21.4% of breast cancer cases have been estimated to be attributable to modifiable risk factors and this proportion may rise up to 34.6% in post-menopausal women [5,6]. This fact has led to an emerging interest in understanding how modifiable risk factors, such as obesity, physical inactivity, and poor dietary patterns, may be related to breast cancer risk [7]. Most of the available studies have focused on the analysis of these factors independently, however a combined approach could be very useful in the development of primary prevention strategies [8]. On the other hand, breast cancer is a heterogeneous disease, of which its natural history and risk factors differ for cases diagnosed before or after menopause. Thus, the need of a stratified approach to better understand the impact of these factors on pre- and post-menopausal breast cancers has been suggested [6,9].

In 2007, the World Cancer Research Fund/American Institute for Cancer Research (WCRF/AICR) released recommendations related to diet, physical activity, and body fat for cancer prevention [10]. Since then, several studies have evaluated the association between the compliance with these recommendations and breast cancer risk. A recent systematic review and meta-analysis reported that adherence to the 2007 WCRF/AICR recommendations was associated with lower risk of breast cancer but with high heterogeneity of study results. This heterogeneity was maintained when the results were stratified by pre- and post-menopausal status and the score was analysed as a categorical variable [11]. Part of this heterogeneity could be explained by the lack of an established standard and operational system to quantify the adherence to the recommendations.

In order to overcome the existing limitations for direct comparability across studies, a guide for research applications has been developed following the updated Third Expert Report for cancer prevention of the WCRF and AICR [12,13]. As far as we know, only two studies—a case-control study and a cohort study focused only on in situ cases of breast cancer—have analyzed the new recommendations and their associations with breast cancer [14,15]. Considering the scarce knowledge on the association between these updated recommendations and invasive breast cancer risk and the inexistence of prospective studies addressing this question, the aim of the present study was to evaluate the association between compliance with the 2018 guidelines of the WCRF/AICR cancer prevention recommendations and the risk of breast cancer in the SUN (“Seguimiento Universidad de Navarra”) Project. 

## 2. Materials and Methods

### 2.1. Study Design and Participants

The SUN Project is a multi-purpose prospective cohort study where all participants are university graduates. The design and methods of this cohort are described in more detail elsewhere [16]. Briefly, it is a dynamic cohort, with continuously open recruitment, designed to evaluate the association between diet (or other lifestyle factors) and chronic diseases. Participants are invited to complete a 556-item baseline questionnaire and those who complete the baseline questionnaire are contacted biennially thereafter and inquired about changes in their lifestyles and incident diseases. Until now, considering from the basal measure and to 18-years-follow up, up to 10 questionnaires have been completed.

**Figure 1:**
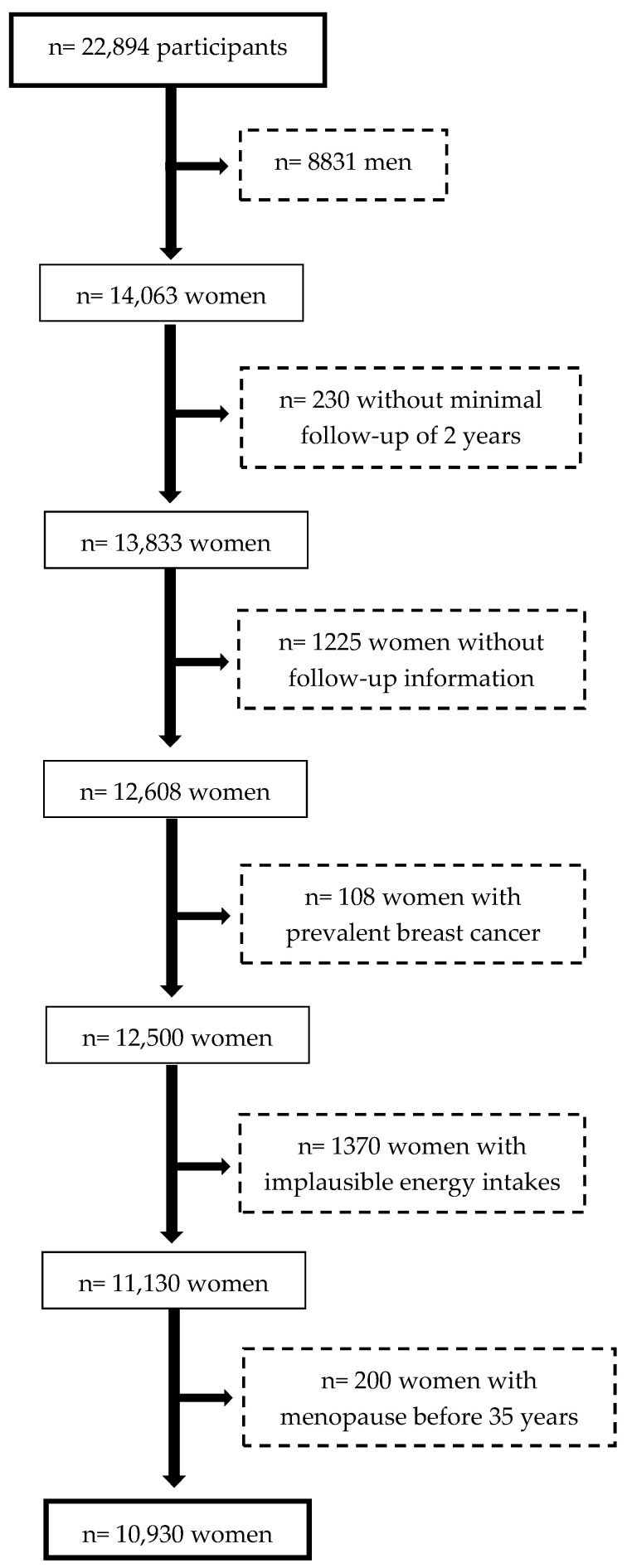
Flow-chart of participants in the SUN (“Seguimiento Universidad de Navarra”) Project.

Up to December 2019, 22,894 participants were recruited and 14,063 of them were female. To allow a minimal follow-up of 2 years, in the present analysis, we only included those participants who were recruited before March 2017. We excluded 1225 participants with no follow-up information (retention 91.4%). Additionally, women with prevalent breast cancer at baseline (n = 108), with energy intakes outside of predefined limits (< 500 or > 3500 kcal/day) [17] (n = 1370), or with self-reported menopause before 35 years of age (n = 200) were also excluded. Figure 1 shows the flow diagram of women finally included in the analytical sample, which consisted of 10,930 participants.

This study was conducted according to the Declaration of Helsinki guidelines. Ethical Approval for this study was provided by the Institutional Review Board of the University of Navarra (approval code 010830; 30 August 2001). All participants included in this study were fully informed about the study objectives and they voluntarily gave informed consent according to the methods approved by our Institutional Review Board.

### 2.2. WCRF/AICR Score Construction

The baseline questionnaire gathered information about medical history, lifestyle, sociodemographic factors, and anthropometric measurements. Physical activity was ascertained at baseline through a validated 17-item questionnaire [18]. In addition, the baseline questionnaire included a previously validated 136-item food-frequency questionnaire (FFQ) [19,20]. Further, the reproducibility of the FFQ was specifically addressed in a subsample of this cohort [21]. To calculate the body mass index (BMI), we used the self-reported weight and height, of which accuracy was previously validated in a subsample of this cohort [22]. The questionnaire also included the question “On average, for how long did you breast-feed your children?”, with four possible answers (nil, <1 month, 1–3 months, >3 months).

The proposed 2018 WCRF/AICR Score included 8 items [13]: (1) having a healthy weight, (2) being physically active, (3) eating a diet rich in wholegrains, vegetables, fruit, and beans, (4) low consumption of fast food and other processed foods, (5) low consumption of red and processed meat, (6) low consumption of sugar-sweetened drinks, (7) low alcohol consumption, and (8) the optional inclusion of exclusive breastfeeding. This last special recommendation was included in an additional analysis since we did not know if breastfeeding was done exclusively (see question about this in the paragraph above). The method of estimating the score is based on the following criteria (Supplementary Table S1): 1 point was assigned for full adherence, 0.5 points for partial adherence, and 0 points when not met. For recommendations with two sub-recommendations, such as consumption of whole grains, vegetables, fruit, and beans recommendation, the scoring weight was divided equally between both to retain a total of one point (0.5, 0.25, and 0 points for meeting, partially meeting, and not meeting each subitem, respectively). Having a healthy weight was also composed of two subitems. Since information on waist circumference was not available at baseline, the value of the BMI was doubled as this is recommended when only one component is available [13].

For the recommendation on “fast food”, we included ultra-processed foods as the total amount of the food items meeting the ultra-processed food category of the NOVA system [23]. Processed meats, sweetened drinks, and alcohol beverages were excluded since they were included as separate items in the scoring system. We divided the caloric intake (kcal/day) from ultra-processed food over the total caloric intake per day and categorized this result into tertiles.

The score of each recommendation was added to obtain the total score. Therefore, the total scores ranged from 0 to 7 points (from 0 to 8 points when breastfeeding was included), with higher values indicating higher compliance with the cancer prevention recommendations. The score was further categorized according to previously defined cut-off points [15]: minimal compliance: ≤3 points; intermediate compliance: >3–≤5 points; and maximum compliance: >5 points.

### 2.3. Incident Breast Cancer Assessment

Incident breast cancer was the primary end point and was considered when a new diagnosis of breast cancer occurred during the follow-up and women were free of breast cancer at the beginning of the study. The new diagnosis of breast cancer was initially self-reported by the participant. Women with a self-reported diagnosis of breast cancer were asked to provide a medical record for confirmation purposes. These documents were evaluated by a trained oncologist who was blinded to exposures of participants and adjudicated the confirmed cases. Moreover, for cases, we also included deaths due to breast cancer reported to the research team by the subject’s next of kin, work associates, and postal authorities or those deaths due to breast cancer identified through the National Death Index, which provides information of the vital status of people living in Spain [24]. The breast cancer diagnosis was confirmed for all the cases, except for those identified using the National Death Index, of which further confirmation was not necessary. Probable cases included both confirmed cases and those breast cancers pending confirmation.

### 2.4. Covariate Assessment

Besides the variables used to build the 2018 WCRF/AICR Score, the baseline questionnaire gathered information on age, years at university, smoke habits, family history of breast cancer, age of menopause (collected in the baseline questionnaire and updated in the 16-year and 18-year follow-up questionnaire), age of menarche, age of first pregnancy, previous or current use of hormone-replacement therapy, use of oral contraceptives, and medication use.

### 2.5. Statistical Analysis

Means and standard deviations were used to summarize the quantitative baseline characteristics of participants and percentages for categorical variables.

To assess the relationship between the 2018 WRCF/AICR recommendations and the risk of breast cancer, we fitted Cox regression models. We estimated Hazard ratios (HR) and their 95% confidence intervals (CI). The score was analyzed as a continuous variable (one-unit increment) and as a categorical variable according to the previously defined cut-off points (≤3 points –lower compliance, reference–, >3–≤5 points, and >5 points). For all analyses, we fitted two multivariable models with successive degrees of adjustment for potential confounders and considered overall breast cancer incidence (regardless of menopausal status) and, then, we did separated analyses according to menopausal status. Selection of confounding variables was based on directed acyclic graphs and information from previous studies. Model 1 was adjusted for age as the underlying time variable and age (in decades) and recruitment period (3 categories) as stratification variables. Model 2 was also adjusted for total energy intake (kcal/day, in tertiles), years at university, smoking status (never smoker/former smoker/current smoker), family history of breast cancer (no/yes, after the age of 45 years/yes, up to the age of 45 years), menopause (yes/no), age at menarche (≤11/12–13/14/≥ 15 years), age at first pregnancy (<25 years and menopausal status/age < 25 years and nulliparous/age ≥ 25 years and nulliparous/first pregnancy before 25 years/first pregnancy being 30 years old or older), use of hormone replacement therapy (yes/no), and oral contraceptive use (yes/no). Analysis for overall breast cancer was additionally adjusted for age at menopause. For post-menopausal women, in addition to age of menopause, model 2 was also adjusted for time since recruitment. Participants with breast cancer, both confirmed and probable cases, were followed up until the date of breast cancer diagnosis, and those without breast cancer until the date of death or last contact. In order to assess the incidence of pre- or post-menopausal breast cancer as an outcome, we split the time at risk of our participants into the time during which they were pre-menopausal and the time during which they were post-menopausal. For women with missing information on age at menopause, we defined post-menopausal status according to the 75th percentile of age at menopause in the study population (52 years of age) [25]. When assessing pre-menopausal breast cancer as the outcome, we excluded those women who reported having had menopause before recruitment and time at risk comprised time since study inception until the age of menopause or when participants turned 52 years of age, whichever occurred first. When assessing post-menopausal breast cancer, time at risk for women who had had their menopause before entering the study started at study inception or when they turned 52 years of age if they had been recruited at an earlier age. For women who were initially pre-menopausal, time at risk started when they turned 52 years of age or at their self-reported age of menopause, whichever occurred last.

Moreover, we estimated the individual association of each component of the 2018 WCRF/AICR score with confirmed breast cancer risk, after adjustment for all other components of the score and the aforementioned potential confounders. All statistical tests were two-sided and statistical significance was set at *p* < 0.05. Statistical analyses were run using the statistical program Stata v.15 (Stata Corp., College Station, TX, USA, 2017).

## 3. Results

A total of 10,930 women were included in the analysis, with a median follow-up of 12.1 years. Table 1 shows the characteristics of the total female participants and their compliance to the 2018 WCRF/AICR recommendations. Participants with higher compliance were older, were more likely to be post-menopausal, to be current smokers, and to have used hormone-replacement therapy, and were less like to be nulliparous.

During 123,297 women’s year of follow-up, we identified 119 confirmed incident cases of breast cancer. When we assessed adherence to the recommendations as a continuous variable, women with higher compliance showed a non-significant lower risk of breast cancer, after adjusting for potential confounders (Table 2). No significant associations were observed for pre- or post-menopausal breast cancer. When we categorized adherence to the recommendations into three categories (minimal compliance: ≤ 3 points; intermediate compliance: >3–≤5 points; and maximum compliance: >5 points), women with a higher adherence to the recommendations showed a non-significant lower risk of overall breast cancer and this association became statistically significant when we considered only post-menopausal breast cancer: a significant association was found in these women with the highest compliance category (> 5 points) compared to the lowest compliance category (≤ 3 points) (HR= 0.27; 95% CI: 0.08-0.93). These estimates were toned down when breastfeeding was included in the analysis (Supplementary Table S2). When probable breast cancers were also included as cases (n = 200 in total), the magnitude of the association of the estimates was weakened (Supplementary Table S3). The HR for the association between overall breast cancer and continuous WCRF/AICR score and overall breast cancer changed from 0.89 (95% CI: 0.69–1.08) to 0.93 (95% CI: 0.78–1.11). For post-menopausal women, comparing the highest compliance category to the lowest category, the HR changed from 0.27 (95% CI: 0.08–0.93) to 0.40 (95% CI: 0.13–1.21), with loss of statistical significance.

The mutually adjusted HRs for the individual components of the 2018 WRCF/AICR score and confirmed breast cancer are shown in Table 3. None of the components of the score were significantly associated with confirmed breast cancer. We did not find any significant associations in the stratified analysis for menopausal status (data not shown).

## 4. Discussion

In the present large prospective study, an inverse association was observed between a higher adherence to the 2018 WCRF/AICR recommendations and incidence of post-menopausal breast cancer. None of the individual components of the score were significantly associated with the risk of breast cancer.

There is growing evidence on the co-occurrence of risk behaviors and their association with chronic diseases such as cancer [26,27]. Thus, comprehensive scores, such as the WCRF/AICR score, which incorporates different indicators, allow the evaluation of an overall healthy lifestyle. A previous meta-analysis of the studies conducted assessing the previous 2007 recommendations reported an inverse association between adherence to the 2007 WCRF/AICR and breast cancer, with moderate-high heterogeneity for the overall study selection [11,14]. Nevertheless, one challenge of the 2007 WCRF/AICR recommendations was the comparability across studies [13] due to the lack of an operational standardized scoring system. Thus, it is interesting to further examine the association between the 2018 WCRF/AICR recommendations and cancer risk in different populations [13].

The few studies whichhave evaluated the association between the updated recommendations and breast cancer are consistent with our results. Turati F et al. [14] conducted a case-control study and found significant inverse associations for overall cases. In the EPIC cohort, significant inverse associations were observed only among women recruited via screening programs and with regular screening participation [15].

With respect to menopausal status, there is more inconsistency. In a previous case-control study conducted by Turati F et al. [14], the association between high adherence to 2018 WRCF/AICR recommendations and breast cancer was observed regardless of menopausal status. We found a protective relationship between higher adherence to the 2018 WCRF/AICR recommendations and invasive breast cancer in post-menopausal women. The observed association may be explained by a healthier profile of plasma biomarkers of inflammation, insulin, and hormonal response with greater adherence to the WCRF/AICR cancer prevention recommendations [28,29]. The lower incidence of breast cancer in post-menopausal women may be due to the different effects of some components of the score according to menopausal status. Thus, the revised 2018 report for breast cancer from WCRF/AIRC indicates that alcohol intake is more strongly associated with breast cancer in post-menopausal women compared with pre-menopausal women (convincing evidence vs. probable evidence) [30]. Also, overweight and obesity are conditions that are penalized in the score; however, they are considered risk factors for post-menopausal breast cancer, but not for pre-menopausal breast cancer [30,31]. Similarly, in the abovementioned meta-analysis on the association between adherence to the 2007 WCRF/AICR recommendations and breast cancer risk, the protective association was significant only for post-menopausal women but not for pre-menopausal women. Nevertheless, there was an unresolved high between-study heterogeneity which could be attributed to the disparity in the cut-off points used to define the highest vs. the lowest category. Thus, and for the sake of comparability, we decided to select external cut-off points based on its previous use with the application of the 2018 WRCF/AICR recommendations [15].

The magnitude of the associations described in our study were toned down when the breastfeeding item was additionally included in the score. Our questionnaire collected any type of breastfeeding and the score recommendation is only referred to the exclusive breastfeeding. Also, the questionnaire only included four possible answers and the cut-off point for the highest category was >3 months. So, this previous result may have been affected by the possible non-differential misclassification produced by the impossibility of distinguishing how much of all breastfeeding time was exclusive and the actual duration of exclusive breastfeeding. Similarly, when probable breast cancers were analysed, the estimated associations were weakened. Again, some degree of misclassification could have happened in these results since unconfirmed diagnoses could include benign breast conditions.

Regarding the specific associations for each individual component, we did not find any association with any of the individual components. Previous evidence about the components related to diet have suggested the importance of overall diet quality as compared to individual food components in breast cancer [32]. Our findings support this and highlight the possible importance of the interaction of components related to diet and other risk factors included in the score. However, we did not disregard the possibility that the lack of association for individual components of the WCRF/AICR lifestyle score in our analyses could be due to the relatively small proportion of breast cancer cases in the study population, especially when we stratified by menopausal status.

Our study has potential limitations. First of all, the SUN Project is largely formed by young people, which could explain the reduced number of incident cases of breast cancer. This could have led to suboptimal statistical power and unstable estimates for some comparisons. However, the age-adjusted standardized incidence was calculated and it was found to be similar to the one described for the Spanish population [33]. Second, our study sample was not representative of the general population. Nevertheless, there is no biological plausibility to expect these results not to be valid for women with a lower educational level. Besides this, the reason for selecting only university graduates was to control for educational level based on restriction. Third, the number of confirmed cases precluded from subgroup comparisons according to breast cancer subtype. Fourth, self-reporting questionnaires could lead to information bias. Nevertheless, in that case, the non-differential misclassification would lead our results toward the null. Moreover, dietary and physical activity information was collected with previously validated questionnaires [18,19,20]. Fifth, information about breast cancer incidence was self-reported and underreporting of cases could have happened. We tried to minimize the possible suboptimal sensitivity with a close follow-up of our participants and the periodical consultation of the National Death Index. Finally, despite adjustment for a range of potential confounders, residual confounding variables might have influenced our results.

Regarding the strengths of our study, we have to point out the prospective nature of the SUN Project, which reduces the possibility of reverse causation bias, the long follow-up of the sample, and the high retention rate. To our knowledge, this is the first prospective study evaluating the association between the 2018 WRCF/AICR recommendations and invasive breast cancer risk. Our results suggest the importance of the approach done in this study, allowing the evaluation of combined possible modifiable risk factors, based on a standardized scoring system to solve the problem found in previous studies on the comparability of the results.

## 5. Conclusions

In conclusion, in this cohort, a protective association was observed between higher compliance of the 2018 WRCF/AICR recommendations and the risk of post-menopausal breast cancer for overall cases. In addition, our results suggested an inverse association of these recommendations with the occurrence of breast cancer, which might be produced by the combined synergistic effects of the diverse individual components. The findings of this study support the advice to comply with WRCF/AICR recommendations to prevent breast cancer among post-menopausal women.

## Figures and Tables

**Table 1:** Baseline characteristics of the female participants in the SUN Project for the overall sample and according to categories of adherence to the 2018 WCRF/AICR recommendation.

	Total n = 10,930	Minimal Compliance n = 817	Intermediate Compliance n = 8521	Maximum Compliance n = 1592	
	0–7 points	≤3 points	3–≤5 points	>5 points	*p*-Value
Age at recruitment (years), mean (SD)	35.0 (10.6)	32.0 (8.9)	34.6 (10.3)	39.9 (11.3)	<0.01
Time of university education (years), mean (SD)	4.8 (1.3)	4.9 (1.3)	4.8 (1.3)	4.8 (1.4)	0.35
Smoking status, n (%) Never Former Current	5646 (51.6) 2491 (22.8) 2794 (25.6)	393 (48.1) 252 (30.8) 172 (21.1)	4403 (51.7) 1980 (23.2) 2138 (25.1)	849 (53.3) 259 (16.3) 484 (30.4)	<0.01
Family history of breast cancer, n (%) No Yes, after the age of 45 years Yes, up to the age of 45 years	9765 (89.3) 947 (8.7) 218 (2.0)	717 (87.7) 84 (10.3) 16 (2.0)	7627 (89.5) 729 (8.6) 165 (1.9)	1421 (89.3) 134 (8.4) 37 (2.3)	0.41
Menopausal status at recruitment, n (%) Pre-menopausal Post-menopausal	10,073 (92.2) 857 (7.8)	789 (96.6) 28 (3.4)	7935 (93.1) 586 (6.9)	1349 (84.7) 243 (15.3)	<0.01
Age at menarche, n (%) ≤11 years 12–13 years 14 years ≥15 years	2211 (20.2) 5981 (54.7) 1852 (16.9) 886 (8.1)	145 (17.7) 445 (54.5) 157 (19.2) 70 (8.6)	1723 (20.2) 4663 (54.7) 1450 (17.0) 685 (8.1)	343 (21.6) 873 (54.8) 245 (15.4) 131 (8.2)	0.18
Age at first pregnancy, n (%) Age < 25 years and nulliparous Age ≥ 25 years and nulliparous First pregnancy before 25 years First pregnancy between 25 and 30 years of age First pregnancy being 30 years old or older	1943 (17.8) 5364 (49.1) 511 (4.7) 1582 (14.4) 1530 (14.0)	185 (22.6) 441 (54.0) 22 (2.7) 80 (9.8) 89 (10.9)	1595 (18.7) 4172 (49.0) 369 (4.3) 1205 (14.1) 1180 (13.9)	163 (10.2) 751 (47.2) 120 (7.5) 297 (18.7) 261 (16.4)	<0.01
Hormone-replacement therapy, n (%) No Yes	10,414 (95.3) 516 (4.7)	800 (97.9) 17 (2.1)	8165 (95.8) 356 (4.2)	1449 (91.0) 143 (9.0)	<0.01
Oral contraceptive use, n (%) No Yes	10,665 (97.6) 265 (2.4)	793 (91.1) 24 (2.9)	8308 (97.5) 213 (2.5)	1564 (98.2) 28 (1.8)	0.13

SD: standard deviation; WCRF/AICR: World Cancer Research Fund/American Institute for Cancer Research.

**Table 2:** Associations between adherence to the WCRF/AICR lifestyle score and overall, pre-menopausal, and post-menopausal confirmed breast cancer risk.

		Overall Breast Cancer		Pre-Menopausal Breast Cancer		Post-Menopausal Breast Cancer	
		Cases/No Cases	HR (95% CI)	Cases/No Cases	HR (95% CI)	Cases/No Cases	HR (95% CI)
**WCRF/AICR score, continuous (0–7) for each additional point**	Model 1*	119/10,811	0.85 (0.68–1.06)	67/9904	0.92 (0.68–1.24)	42/3256	**0.68 (0.47–0.99)**
	Model 2**	119/10,811	0.89 (0.69–1.08)	67/9904	0.94 (0.69–1.27)	42/3256	0.74 (0.51–1.06)
**WCRF/AICR score:** ≤ 3 > 3.00-≤ 5.00 > 5.00	Model 1*	9/808 94/8427 16/1576	1.00 0.81 (0.41–1.61) 0.55 (0.24–1.27)	3/786 57/7878 7/1342	1.00^a^ 1.00^a^ 0.66 (0.30–1.45)	5/164 31/2494 6/759	1.00 **0.31 (0.12–0.81)** **0.18 (0.05–0.59)**
≤ 3 > 3.00-≤ 5.00 > 5.00	Model 2**	9/808 94/8427 16/1576	1.00 0.87 (0.44–1.74) 0.62 (0.27–1.43)	3/786 57/7878 7/1342	1.00^a^ 1.00^a^ 0.67 (0.30–1.47)	5/164 31/2494 6/759	1.00 0.45 (0.16–1.23) **0.27 (0.08–0.93)**

* Model 1: age as underlying time variable in all analyses and all analyses stratified by age (in decades) and recruitment period. ** Model 2: additionally adjusted for total energy (in tertiles), years at university, smoking status (never/former/current), family history of breast cancer (no/yes, after the age of 45 years/yes, up to the age of 45 years), age at menarche (≤ 11 years/12-13/14/≥ 15 years), age at first pregnancy (< 25 years and menopausal status/age < 25 years and nulliparous/age ≥ 25 years and nulliparous/first pregnancy before 25 years/first pregnancy being 30 years old or older), hormone-replacement therapy (yes/no), oral contraceptive use (yes/no), and menopausal status (except in models stratified by menopausal status), and age at menopause (except in models with pre-menopausal women). Models for post-menopausal breast cancer were also adjusted for time since recruitment. ^a^ Due to the small sample size of the reference category, the lowest category and the intermediate category were merged together and considered as the reference category.

**Table 3:** Associations between adherence to individual components of the WCRF/AICR score and overall breast cancer risk.

	Total Study Population		
Component of the Score	Cases/No Cases	HR (95% CI)^a^	p-Trend
Be in a healthy weight 0 0.5 1	9/881 16/1391 94/8539	1.00 0.72 (0.31–1.64) 0.90 (0.45–1.79)	0.85
Be physically active 0 0.5 1	13/844 20/1301 86/8666	1.00 0.96 (0.47–1.94) 0.65 (0.36–1.19)	0.07
Eat whole grains, vegetables, fruit, and beans 0–0.25 0.5 0.75–1	7/637 7/713 105/9461	1.00 1.02 (0.34–2.98) 0.64 (0.26–1.54)	0.17
Limit “fast foods” 0 0.5 1	30/3613 47/3596 42/3602	1.00 1.47 (0.92–2.34) 1.11 (0.67–1.82)	0.77
Limit red and processed meat 0 0.5 1	110/9953 7/681 2/177	1.000.91 (0.41–2.01) 1.17 (0.28–4.90)	0.99
Limit sugary drinks 0 0.5 1	2/291 79/7569 38/2951	1.00_b_ 1.00^b^ 0.93 (0.62–1.40)	-
Limit alcohol 0 0.5 1	10/623 79/7267 30/2921	1.00 0.93 (0.48–1.83) 0.89 (0.43–1.87)	0.76

^a^ Adjusted for age (underlying time variable), total energy (in tertiles), years at university, smoking status (never/former/current), family history of breast cancer (no/yes, after the age of 45 years/yes, up to the age of 45 years), age at menarche (<10 years/≥10 to ≤15 years/>15 years), age at first pregnancy (<25 years and menopausal status/age < 25 years and nulliparous/age ≥ 25 years and nulliparous/first pregnancy before 25 years/first pregnancy being 30 years old or older), hormone-replacement therapy (yes/no), oral contraceptive use (yes/no), menopausal status, and age at menopause. Additionally, all individual components were adjusted for the remaining components of the WCRF/AICR lifestyle score. ^b^ Due to the small sample size of the reference category, the lowest category and the intermediate category were merged together and considered as the reference category.

## Supplementary Materials

The following are available online at https://www.mdpi.com/2072-6643/12/7/2076/s1. Table S1: Operationalization of the 2018 WRCF/AICR Cancer Prevention Recommendations in the SUN cohort; Table S2: Associations between the WCRF/AICR lifestyle score and overall breast cancer risk, stratified by menopausal status, including breastfeeding; Table S3: Associations between the WCRF/AICR lifestyle score and the risk of overall probable breast cancer, stratified by menopausal status.
